# Identification of Malignant Cell Populations Associated with Poor Prognosis in High-Grade Serous Ovarian Cancer Using Single-Cell RNA Sequencing

**DOI:** 10.3390/cancers14153580

**Published:** 2022-07-22

**Authors:** Naoki Sumitani, Kyoso Ishida, Kenjiro Sawada, Tadashi Kimura, Yasufumi Kaneda, Keisuke Nimura

**Affiliations:** 1 Division of Gene Therapy Science, Department of Genome Biology, Osaka University Graduate School of Medicine, Suita, Osaka 565-0871, Japan; u345870f@ecs.osaka-u.ac.jp (N.S.); ks-ishida@gyne.med.osaka-u.ac.jp (K.I.); kaneday@gts.med.osaka-u.ac.jp (Y.K.); 2 Department of Obstetrics and Gynecology, Osaka University Graduate School of Medicine, Suita, Osaka 565-0871, Japan; daasawada@gyne.med.osaka-u.ac.jp (K.S.); tadashi@gyne.med.osaka-u.ac.jp (T.K.)

**Keywords:** ovarian cancer, cancer stem cell, CTL, CA125

## Abstract

**Simple Summary:**

Ovarian cancer has a high recurrence rate (~75%), and tumor heterogeneity is associated with such tumor recurrence. However, it is still poorly understood in ovarian cancer. To reveal tumor heterogeneity, we performed single-cell RNA sequencing (RNA-seq) of serous ovarian cancer cells from four different patients: two with primary carcinoma, one with recurrent carcinoma, and one with carcinoma treated with interval debulking surgery. As a result, we found two malignant tumor cell subtypes associated with poor prognosis. One malignant population included the earliest cancer cells and cancer stem-like cells. SLC3A1 and PEG10 were identified as the marker genes of cancer-initiating cells. The other malignant population expressing CA125 (MUC16), the most common biomarker for ovarian cancer, is associated with a decrease in the number of tumor-infiltrating cytotoxic T lymphocytes (CTLs). Our findings will offer new markers for diagnosis and choosing treatments targeting the malignant populations in ovarian cancer.

**Abstract:**

To reveal tumor heterogeneity in ovarian cancer, we performed single-cell RNA sequencing (RNA-seq). We obtained The Cancer Genome Atlas (TCGA) survival data and TCGA gene expression data for a Kaplan–Meier plot showing the association of each tumor population with poor prognosis. As a result, we found two malignant tumor cell subtypes associated with poor prognosis. Next, we performed trajectory analysis using scVelo and Monocle3 and cell–cell interaction analysis using CellphoneDB. We found that one malignant population included the earliest cancer cells and cancer stem-like cells. Furthermore, we identified SLC3A1 and PEG10 as the marker genes of cancer-initiating cells. The other malignant population expressing CA125 (MUC16) is associated with a decrease in the number of tumor-infiltrating cytotoxic T lymphocytes (CTLs). Moreover, cell–cell interaction analysis implied that interactions mediated by LGALS9 and GAS6, expressed by this malignant population, caused the CTL suppression. The results of this study suggest that two tumor cell populations, including a cancer-initiating cell population and a population expressing CA125, survive the initial treatment and suppress antitumor immunity, respectively, and are associated with poor prognosis. Our findings offer a new understanding of ovarian cancer heterogeneity and will aid in the development of diagnostic tools and treatments.

## 1. Introduction

Ovarian cancer is the eighth highest cause of death among all cancers and accounts for 4.7% of the total cancer deaths in women [1]. Approximately 310,000 women are diagnosed with ovarian cancer annually, and 210,000 die in a year [1]. Patients with early-stage ovarian cancer show good prognosis (five-year relative survival rate 92%), whereas advanced-stage ovarian cancer leads to poor prognosis (five-year survival rate 29%) [2]. Unfortunately, ~75% of patients are diagnosed in the advanced stage [2]. One reason why ovarian cancer is difficult to detect in the early stage is that the symptoms (such as abdominal bloating and early satiety) are not specific to ovarian cancer [3]. Another reason is that no practical examinations are available for detecting ovarian cancer in the early stage [4]. Cancer antigen 125 (CA125) is known as the most common tumor marker for ovarian cancer [5] and is usually used to assess the response to treatment in patients with ovarian cancer [6]. However, because CA125 often does not increase in the early stage of ovarian cancer and it also increases in other prevalent benign diseases, screening tests using CA125 cannot reduce ovarian cancer mortality [7]. There is a need to understand more about tumor progression by revealing tumors’ initial properties and how they have progressed or recurred.

Among ovarian cancer patients, 70 to 75% develop recurrence [8], following chronical relapse with increasing chemoresistance [9,10]. A single HGSOC patient has multiple distinct tumor immune microenvironments, and such intra-tumor heterogeneity is the leading cause of drug resistance and recurrence, causing poor prognosis [11,12,13]. In recent years, many studies using single-cell RNA sequencing (scRNA-seq) have revealed the intra-tumor heterogeneity of ovarian cancer. Shih et al. (2018) found that metastatic tumors require fewer epithelial tumor cells to maintain the tumor microenvironment than primary tumors [14]. Zhao et al. (2021) reported a tumor subpopulation expressing IFI6 as being pivotal in ovarian cancer carcinogenesis [15]. In the same study, they also found that IFI6 was involved in cisplatin resistance through the NF-κB pathway. However, the type of epithelial cells in the tumor that are associated with poor prognosis and the mechanism by which they lead to poor prognosis remain poorly understood.

Of all the ovarian cancers, serous ovarian cancer is the most common, accounting for ~50% of ovarian cancers [14]. In particular, high-grade serous carcinoma, accounting for ~45% of all cases, is the deadliest ovarian cancer [16]. In the present study, we performed scRNA-seq of serous ovarian cancer cells from four patients and identified two malignant tumor subtypes associated with poor prognosis. One malignant population (C0) included the earliest cancer cells and cancer stem-like cells, which survived initial treatment and proliferated again. Additionally, we identified SLC3A1 and PEG10 as the marker genes of the earliest cancer cells. These genes may be associated with tumorigenesis or recurrence. The other malignant population (C4) was associated with a decrease in tumor-infiltrating cytotoxic T lymphocyte (CTL) population. C4 expressed higher levels of LGALS9 and GAS6 than other cancer cells and interacted with immune cells via these molecules. Both LGALS9 and GAS6 are involved in the decrease in the number of CTLs and serve as potential targets for treating ovarian cancer. Our findings will help to improve the understanding of ovarian cancer heterogeneity and aid in the development of diagnostic markers and therapies.

## 2. Materials and Methods

### 2.1. Construction of scRNA-seq and RNA-seq Libraries from Ovarian Cancer Specimens

We collected specimens from patients who had received surgical treatment for high-grade serous ovarian cancer at Osaka University Hospital and had given consent. The experiment was approved by the Osaka University Ethics Committee (approval number 709). The corrected specimens were finely minced using scissors after removing the non-tumor tissue. The minced tumor was incubated in 0.1% collagenase (034-17604, FUJIFILM, Tokyo, Japan) and 2% fetal bovine serum (FBS; 172012, Sigma-Aldrich, St. Louis, MO, USA) in PBS at 37 °C for 1 h. The tumor suspension was filtered with a 70 µm cell strainer (352350, Falcon, Hongkong, China) and then washed with 2% FBS/PBS twice. Dead cells and debris were removed using Ficoll–Paque premium (17544203, Cytiva, Marlborough, MA, USA). The total RNA was obtained from 5 × 10^6^ cells for RNA-seq using ISOGEN (311-02501; Nippon Gene Co., Ltd., Tokyo, Japan) and ethachinmate (312-01791; Nippon Gene Co., Ltd.). Thereafter, 1–5 × 10^6^ cells were cryopreserved in CELLBANKER 1 (CB011, NIPPON ZENYAKU KOGYO Co., Ltd., Koriyama City, Japan) in liquid nitrogen for scRNA-seq. RNA-seq libraries were generated from 1 µg of RNA using NEBNext Poly(A) mRNA Magnetic Isolation Module (#E7490, New England BioLabs, Ipswich, MA, USA) and NEBNext Ultra RNA Library Prep Kits for Illumina (#E7530, NEW ENGLAND BioLabs, Ipswich, MA, USA) as previously described [17]. To generate scRNA-seq libraries, the cryopreserved samples were thawed and suspended as 1 × 10^6^ cells/mL in 0.04% bovine serum albumin (BSA; A9418, Sigma-Aldrich, St. Louis, MO, USA)/PBS. Cells were stained with propidium iodide (PI; 34-07881, DOJINDO, Kumamoto, Japan), and the PI-negative cells were sorted using FACS Aria III. After washing the sorted cells with 0.04% BSA/PBS, cells were suspended as 1000 cells/µL in 0.04% BSA/PBS. The scRNA-seq libraries were generated using Chromium Single Cell 3′ Reagent Kit (10x Genomics) and by setting the target cell recovery to 10,000. The sequencing libraries were analyzed using HiSeq X Ten.

### 2.2. Bioinformatics

The following pieces of software were used in the study:

CellPhoneDB Python package 2.1.7 [18,19]

Cell Ranger 3.1.0 (10 X GENOMICS)

CIBERSORTx. Available online: https://cibersortx.stanford.edu (accessed on 21 May 2021) [20]

clusterProfiler R package 3.14.3

corrplot R package 0.90

DESeq2 R package 1.26.0 [21]

dplyr R package 1.0.5

enrichplot R package 1.6.1

infercnv R package 1.2.1. Available online: https://www.bioconductor.org/packages/release/bioc/html/infercnv.html) (accessed on 16 April 2021)

ggplot2 R package 3.3.3

gplots R package 3.1.1

GSVA R package 1.34.0 [22]

loompy Python package 3.0.6

Monocle 3 0.2.3.3 [23,24,25]

org.Hs.eg.db R package 3.10.0

Python 2.7.15 and 3.6.12. Available online: https://www.python.org (accessed on 2 February 2021)

R 3.6.3 and 4.0.5. Available online: https://www.r-project.org (accessed on 21 October 2020)

RStudio 1.4.1103 and 1.1.456. Available online: https://www.rstudio.com (accessed on 21 October 2020)

Samtools 1.9

scrublet Python package 0.2.3 [26]

scvelo Python package 0.2.3 [27,28,29]

Seurat R package 3.2.3, 4.0.3 [30,31,32,33]

SeuratDisk R package 0.0.0.9019

SeuratWrappers R package 0.3.0

STAR 2.5.3a_modified

stringr R package 1.4.0

StringTie 1.3.4b

survival R package 3.2-10

velocyto Python package 0.17.17 [28]

#### 2.2.1. scRNA-seq Data Analysis

Cell Ranger was used to obtain gene expression count matrices from BCL files from four single-cell libraries (Supplementary Table S1). The obtained data were analyzed with Seurat. To remove cells with low quality, cells were filtered with the following options: subset = nFeature_RNA > 200, nFeature_RNA < 11,000 and percent.mt < 75. Finally, a total of 33,591 cells remained. The data were normalized, scaled, and integrated using 20 dimensions. We reduced dimensions of the merged data with principal component analysis (PCA) and uniform manifold approximation and projection (UMAP) and clustered the data with the following parameters: npcs (total number of PCs to compute and store) set to 30; reduction set to PCA; dims set to 1:20; and resolution set to 0.5. Consequently, we identified 20 clusters. Gene expression count matrices were analyzed with scrublet to identify doublets in single-cell libraries. Two hundred and seventy-eight cells were doublets out of 33,591 cells in 20 clusters. Gene set enrichment analysis (GSEA) was performed for tumor clusters with the clusterProfiler R package using the marker genes that were expressed at a higher level in each tumor cluster than in the other tumor clusters. The marker genes were identified using Findmarkers of the Seurat R package. We obtained The Cancer Genome Atlas (TCGA) survival data and TCGA gene expression data via cBioPortal [34,35] for the Kaplan–Meier plot. In each plot, patients in the top 30% and bottom 30% for the scaled expression of the marker genes or the proportion of malignant tumor cluster (C0 or C4 cluster) were compared with survival R package. The marker genes of each tumor cluster were the genes expressed at a higher level in the tumor cluster than in the other clusters, including non-tumor clusters.

##### 2.2.2. Bulk RNA-seq Data Analysis

Paired-end reads in the form of FASTQ files from 25 RNA-seq libraries of tumors or ascites were mapped to the reference human genome hg19 by STAR (Supplementary Table S2). We included other types of ovarian cancer, including clear cell carcinoma and endometrioid, serous, and mucinous carcinoma, to examine the properties of HGSOC. Mapped data in the form of SAM files were converted into BAM files using Samtools. From the BAM files, gene expression levels were calculated using StringTie. We used the gtf file based on hg19 from illumina igenomes as the reference. The obtained data were analyzed with DESeq2 to get gene expression count matrix with genes corresponding to rows and samples corresponding to columns. The matrix was analyzed with GSVA to calculate the signature scores of C0 and C4 for each sample. The marker genes for C0 or C4 were used as gene sets.

##### 2.2.3. Digital Cytometry Analysis

Bulk RNA-seq expression data of tumors and ascites were used to calculate the cell proportion with CIBERSORTx. The cells were classified into 19 Seurat clusters (the Dividing tumor S cluster and Dividing tumor G2M cluster were integrated into the Dividing tumor cluster). The Hclust R package was used to compute the hierarchical clustering, with the parameter “method” set to “ward.D”.

TCGA RNA-seq expression data were used to calculate the cell proportion in the tumors with CIBERSORTx. The cells were classified into 19 Seurat clusters, except the Unknown cluster. The Hclust R package was used to compute hierarchical clustering, with the parameter “method” set to “complete”.

##### 2.2.4. Copy Number Variation (CNV) Analysis

The single-cell expression count matrix of 33,591 cells was analyzed with the infercnv R package to calculate chromosomal CNV. We used blood cells and endothelial cells as reference cells. The CNV score of each cell was defined as the sum of {(Gene CNV Score of the cell) − 1)}^2^ [36].

##### 2.2.5. Single-Cell Trajectory Analysis

To measure the RNA velocity of single cells, the cellranger output folder was processed on the command line using the velocity python package to prepare loom files. Loom files of four samples were merged using the loompy python package. The merged loom file contained 33,927 cells, including all 33,591 cells analyzed in Seurat. The loom file was read in R and converted into Seurat object using the SeuratWrappers R package. From the object, we selected the 15,603 cells in tumor clusters and converted them into h5ad files using the SeuratDisk R package. The H5ad files were analyzed using the scvelo python package to calculate the latent time of each tumor cell.

Single-cell pseudotime trajectory analysis was performed with monocle3. Only the cells in tumor clusters were analyzed. Root was determined by the mean expression levels of PEG10 and SLC3A1, which were identified as the marker genes of the oldest tumor cells in latent time analysis.

##### 2.2.6. Cell–Cell Interaction Analysis

Normalized count data for scRNA-seq and meta files of cell type annotation were analyzed with CellPhoneDB to estimate cell–cell interaction levels between Seurat clusters. The default database and parameters were used.

## 3. Results

### 3.1. Identification of Malignant Tumor Clusters Associated with Poor Prognosis

**Figure 1:**
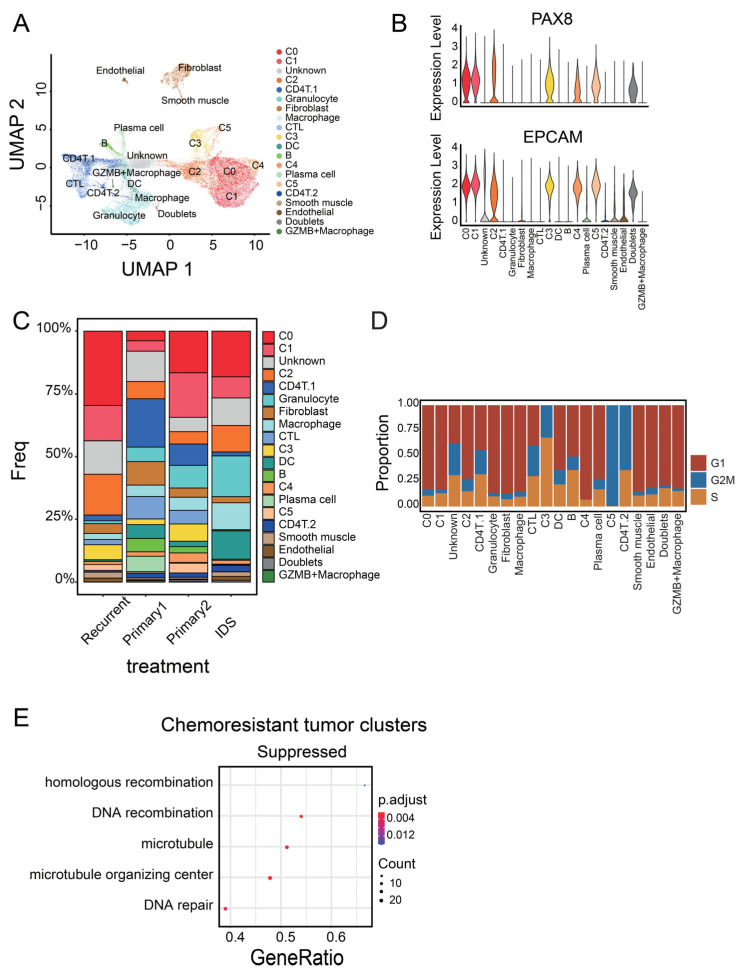
Identification of tumor cell populations associated with poor prognosis. (**A**) UMAP of 33,591 ovarian cancer cells from four patients, colored by cluster. (**B**) Violin plots of marker gene expression in ovarian epithelial tumor cells in Seurat clusters. (**C**) Stacked bar graph showing the cell proportion of Seurat clusters in four patients; 19 clusters were present in all four patients, while the GZMB+ macrophage cluster was present only in three patients. (**D**) Stacked bar graph of cell cycle in Seurat clusters. Each cell was classified into G1, S, or G2M based on the gene expression level. (**E**) GSEA dot plot for chemoresistant tumor clusters (C0, C1, C2, and C4). They were compared with chemosensitive tumor clusters (C3 and C5).

To classify epithelial ovarian cancer cells and identify subpopulations common in patients, we divided cancer cells based on their gene expression level. We analyzed cancer cells from four different samples: two primary carcinomas, one recurrent carcinoma, and one carcinoma from a patient treated with interval debulking surgery (IDS). IDS is the standard treatment for ovarian cancer, in which we performed chemotherapy twice before and after surgery [37]. We can obtain cancer cells after chemotherapy from the sample, which is useful to find out chemosensitive cell populations. Cells from the four patients were integrated and mixed (Supplementary Figure S1A and Table S1). To use as many cells as possible, we excluded low-quality cells with a percent.mt < 75 (Supplementary Figure S1B). After removing low-quality cells, 33,591 cells were integrated and divided into 20 clusters based on scaled gene expression levels (Figure 1A). We determined the cell types of all clusters based on marker gene expression profiles. We identified six epithelial tumor clusters expressing PAX8 [38] and EPCAM [39] (C0–C5, Figure 1B). In addition, we identified three T-cell clusters expressing CD3E, two B-cell clusters expressing CD79A, a granulocyte cluster expressing S100A8, macrophage clusters expressing ITGAM, a dendritic cell (DC) cluster expressing ITGAX, a fibroblast cluster expressing FBLN2, a smooth muscle cluster expressing SYNPO2, and an endothelial cell cluster expressing VWF (Supplementary Figure S1C). We found a small cluster that expressed the marker gene for hematopoietic cells (PTPRC) and that for cancer cells (PAX8) (Supplementary Figure S1B,D). Most of the cells in the cluster were doublets. Moreover, we found another small macrophage cluster expressing GZMB (Supplementary Figure S1D). For subdivision, T cells expressing CD8A were regarded as CD8+ T cells (CTLs), and the other two T-cell clusters expressing CD4 were classified into CD4+ T-cell clusters (Supplementary Figure S1E). CD4T.1, expressing FOXP3, contains regulatory T cells (Tregs, Supplementary Figure S1E). In the same way, we classified B cells expressing SDC1 as plasma cells and the other cluster as the B-cell cluster (Supplementary Figure S1E). We excluded the doublets and unknown clusters including a number of low-quality cells from further analysis. Investigation of the cell proportion in the four samples revealed that all patients displayed the six tumor clusters (Figure 1C). Ultimately, we found that the six tumor clusters (C0–C5) were common to all patients.

To exclude chemosensitive tumor clusters, we performed cell cycle analysis. We classified the phase of each cell into G1, S, or G2 based on the gene expression levels. C3 and C5 mostly comprised cells in the S and G2M phases, respectively (Figure 1D). The other tumor clusters were mainly composed of cells in the G1 phase. Chemotherapy, a standard therapy for ovarian cancer, is more effective for dividing cancer cells than non-dividing cells. In fact, the proportion of C3 and C5 was remarkably low in patients treated with IDS (Figure 1C). These data suggest that C3 and C5 may be the main targets of chemotherapy, and malignant clusters may be found in the four other tumor clusters (C0–C2 and C4).

To confirm whether C3 and C5 clusters are the main targets of chemotherapy, we performed GSEA. Cancer cells belonging to C0, C1, C2, and C4 had low expression of genes related to the microtubule, microtubule organizing center, and DNA repair (Figure 1E, *p* = 0.005333952, 0.002006465, and 0.003599955, respectively, Supplementary Figure S2A). These pathways are the common targets of anticancer drugs, such as taxane and carboplatin, though DNA repair may also help to overcome genotoxic stress from chemotherapy [40,41]. In addition, C0 expressed genes related to the negative regulation of programmed cell death (*p* = 0.03556543, Supplementary Figure S2B). This result suggests that these four clusters were more likely to survive the chemotherapy, leading to a poor prognosis.

### 3.2. Identifying Malignant Tumor Cluster Properties

**Figure 2:**
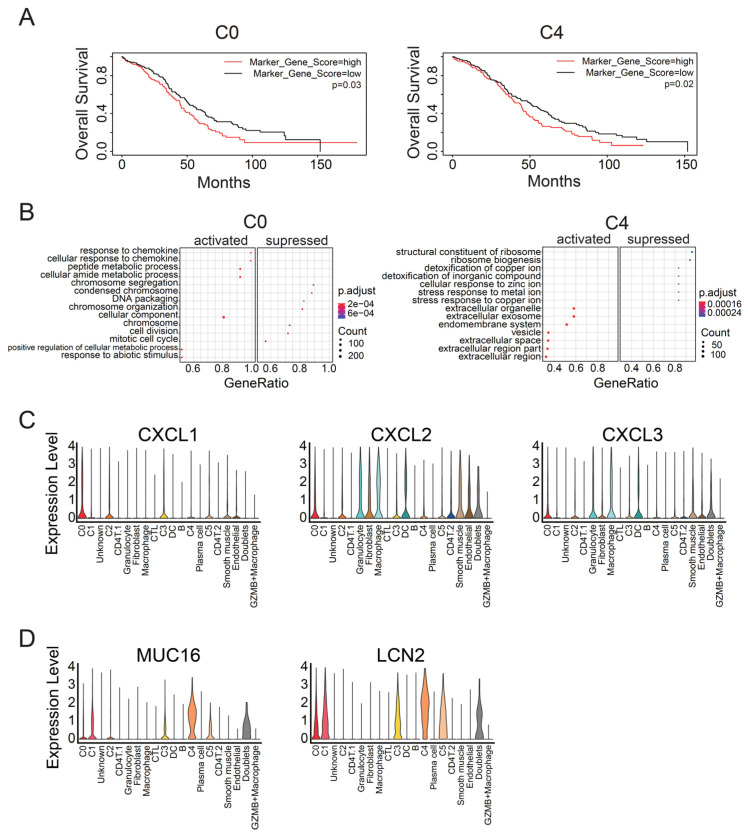
GSEA of C0 and C4 clusters, the two malignant cell populations. (**A**) Kaplan–Meier plots for the C0 and C4 clusters, the two tumor clusters associated with poor prognosis, using TCGA survival data. The *p*-value was calculated using the log-rank test. (**B**) GSEA dot plots for the two malignant tumor clusters. Each tumor cluster was compared with the other tumor clusters. Each plot displays seven activated categories and seven suppressed categories. Dot color indicates adjusted p-value, and dot size indicates gene counts enriched to the Gene Ontology (GO) term. (**C**) Violin plots of chemokine expression in each Seurat cluster. (**D**) Violin plots showing the expression levels of the representative genes for the C4 cluster.

To identify malignant tumor clusters associated with poor prognosis in the four tumor clusters (C0–C2 and C4), we compared the overall survival between patients with a high or low expression level of marker genes in each cluster. We calculated the z-score of the representative genes in each cluster for TCGA samples. Patients with high z-scores of clusters C0 or C4 showed poorer prognoses than those with low scores (*p* = 0.03 and 0.02, respectively; Figure 2A). In contrast, C1 and C2 did not significantly affect the survival period (*p* = 0.8 and 0.4, respectively, Supplementary Figure S3). The other clusters, including non-tumor clusters, also did not significantly affect the survival period (Supplementary Figure S3). C0 and C4 did not express ACTA2 or VIM, the marker genes for cancer-associated fibroblasts (CAFs) [42], and expressed IFI6, which is associated with a malignant subpopulation in ovarian cancer [15], at a comparable level to other tumor clusters (Supplementary Figure S4). However, C0 and C4 had their own marker genes and expressed these genes at higher levels than the other clusters (Supplementary Figure S5A). Indeed, we detected C0- and C4-like clusters in other single-cell data by calculating the expression levels of these marker genes for each cell (cluster 17 and cluster 12, respectively, Supplementary Figure S5B) [43]. These data suggest that C0 and C4 are the malignant tumor clusters associated with poor prognosis in ovarian cancer.

To identify the mechanisms by which C0 and C4 clusters led to poor prognosis, we performed GSEA for C0 and C4. Cancer cells belonging to C0 expressed genes related to the response to chemokine, in contrast to cells of the other tumor clusters (Figure 2B). Regarding the expression of specific genes, C0 exhibited high expression of CXCL1, CXCL2, and CXCL3 (Figure 2C). In contrast, C4 expressed genes related to the release of extracellular vesicles (Figure 2B). Furthermore, C4 significantly expressed CA125 (MUC16) and LCN2 (Figure 2D). This result implies that C4 is involved in tumor malignancy, since CA125 is a common diagnostic marker for monitoring patients with ovarian cancer and the increase in CA125 level is generally associated with drug resistance and tumor progression [44].

**Figure 3:**
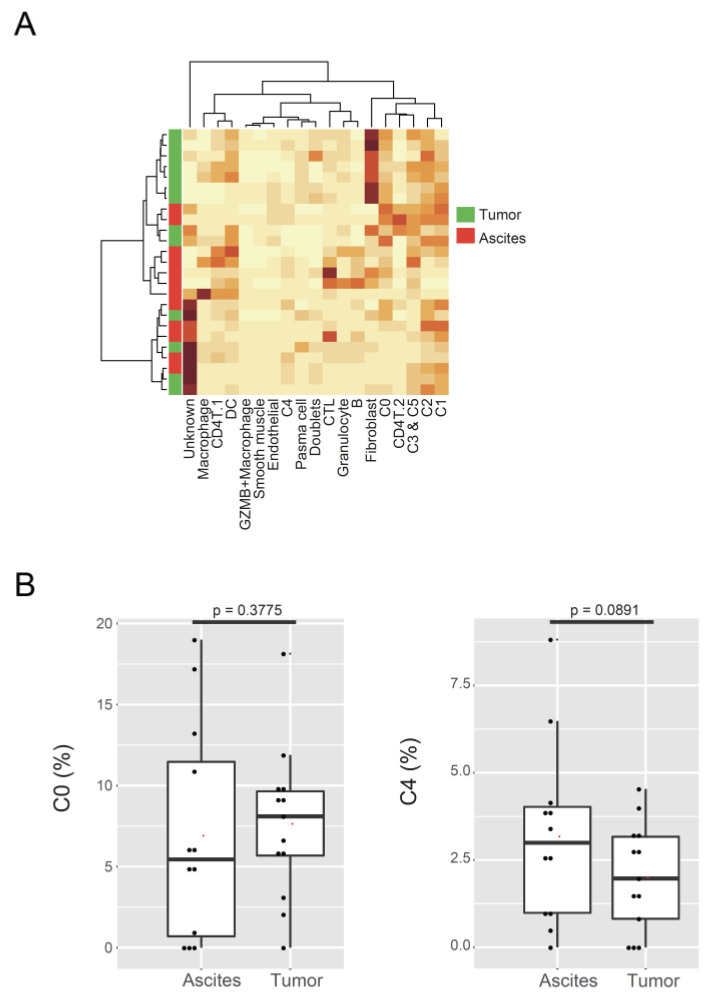
Localization of malignant tumor populations in ascites. (**A**) Heatmap of Seurat cluster proportions in 25 samples of tumors or ascites, calculated using CIBERSORTx. (**B**) Box plots showing the proportions of the C0 and C4 clusters in tumor or ascites.

As CA125 (MUC16) is known to facilitate peritoneal metastasis by binding mesothelin [45], we examined the cell proportion of the two malignant clusters in tumor and ascites. Bulk RNA-seq data of 25 samples (12 from ascites and 13 from tumor) were used to calculate the proportion of each cluster (Figure 3A, Supplementary Table S2). The proportion of C0 and C4 clusters showed no significant difference in tumor or ascites (C0, *p* = 0.3775; C4, *p* = 0.0891; Figure 3B). We substituted cell proportion with GSVA score, using the marker genes for C0 or C4 as gene sets, and produced the same results (C0, *p* = 0.3548; C4, *p* = 0.0998; Supplementary Figure S6). These data suggest that the two malignant clusters existed both in tumors and ascites in comparable proportions.

**Figure 4:**
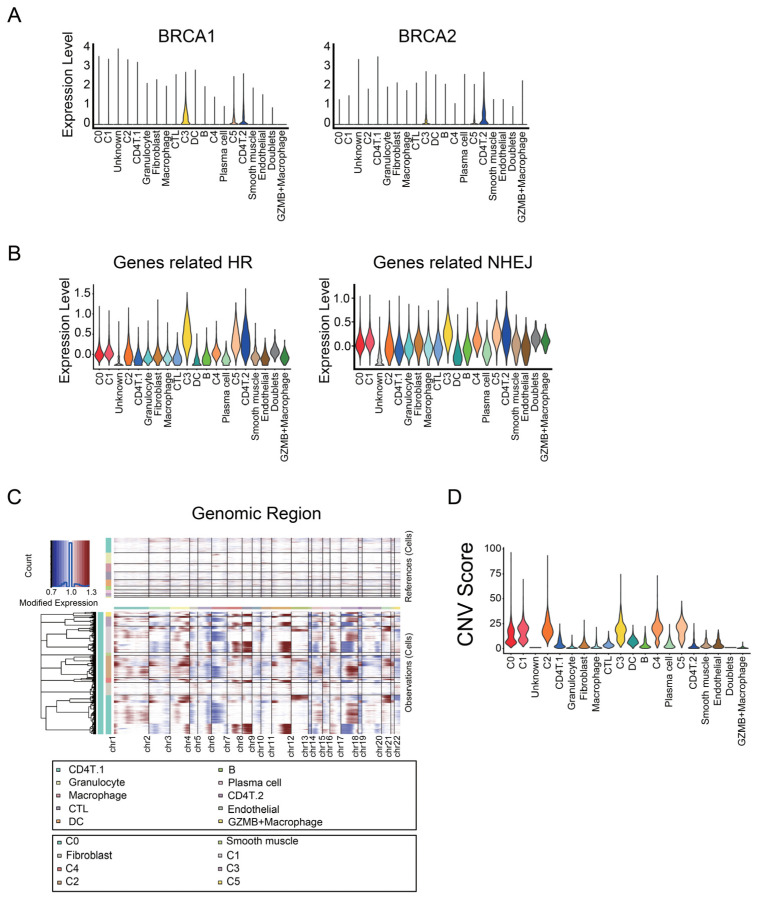
Genome instability of malignant tumor populations. (**A**) Violin plots showing the expression level of BRCA1 and BRCA2 in Seurat clusters. (**B**) Violin plots showing the average expression levels of genes related to homologous recombination (HR) and non-homologous end-joining (NHEJ). (**C**) Heatmap of CNVs on chromosomes (cols) for individual cells (rows), estimated using the infercnv R package. Blood cells and endothelial cells were used as references for estimation. (**D**) Violin plot of the CNV score of the cells in Seurat clusters. The means of CNV scores in the two malignant tumor clusters were slightly lower than or almost the same as those in the other tumor clusters.

To investigate the difference in homologous recombination deficiency (HRD) status between samples or clusters, we checked the expression levels of genes related to HR or non-homologous end-joining (NHEJ). C3 and C5, the dividing cancer cells, expressed BRCA1 and BRCA2, whose variant and insufficiency lead to HRD (Figure 4A). On the other hand, cancer cells belonging to C0, C1, and C4 had lower expression levels of HR-related genes, including BRCA1 and BRCA2 (Figure 4B).

Next, we examined whether C0 and C4 clusters had chromosomal instability. We inferred CNVs of cancer cells using the infercnv R package (Figure 4C). In the same clusters, cancer cells had a different CNV pattern by patients (Supplementary Figure S7). From the output data processed with infercnv, the CNV score of each cell was calculated to evaluate HRD. The mean CNV scores in C0, C1, C2, C3, C4, and C5 were 13.54664, 16.96137, 20.43466, 18.3082, 19.89653, and 19.26681, respectively (Figure 4D). Although the CNV-based risk score correlates with the overall survival in ovarian cancer [46], the CNV scores in C0 and C4 were slightly lower than or almost the same as those in the other tumor clusters. These data suggest that chromosomal instability may not be associated with the two malignant tumor clusters related to poor prognosis.

### 3.3. C0 Cluster Includes the Oldest Epithelial Cancer Cells and Cancer Stem-Like Cells

**Figure 5:**
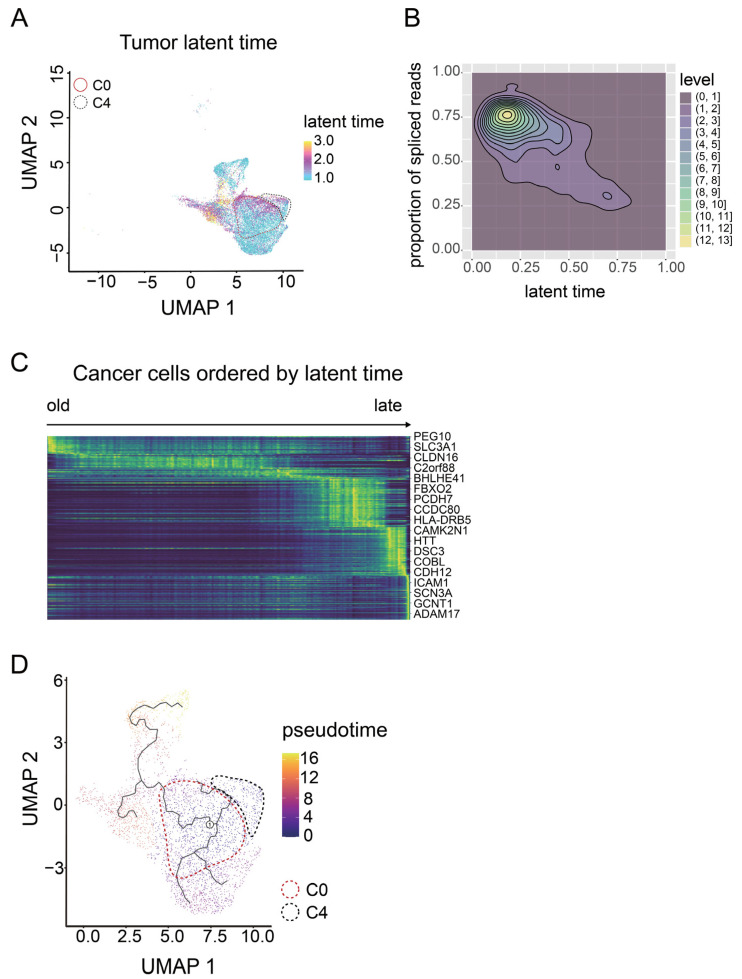
Pseudotime analysis of malignant tumor populations. (**A**) UMAP of the cells in tumor clusters; color represents the latent time. Latent time was calculated using the scvelo python package. (**B**) Contour plot showing the relation between latent time and percentage of spliced reads. (**C**) Heatmap of genes (rows) for 15,603 cancer cells (cols) ordered by latent time. (**D**) UMAP of cells in tumor clusters, colored by pseudotime, calculated using Monocle3.

We next sought to identify the cell type of tumor cells in the earliest stage or cancer stem-like cells. Since conventional markers of cancer stem cells, namely CD133, CD44, KIT, and ALDH1A1 [47], were expressed in all the tumor clusters to a similar extent (Supplementary Figure S8), we performed cell trajectory analysis. First, we calculated each cell’s latent time, which is a measure of cell progress based on transcription and splicing dynamics, unlike existing similarity-based pseudotime (Figure 5A) [27]. Unspliced reads would be spliced as time went on. Thus, the cells including more spliced reads were linked to the longer latent time. (Figure 5B). After sorting cancer cells by latent time, PEG10 and SCL3A1 were listed as the marker genes for the oldest cancer cells (Figure 5C). Thereafter, we calculated the pseudotime, determining the root of cancer cells based on the expression level of PEG10 and SCL3A1. The earliest cells were included in the C0 cluster, while the other malignant tumor clusters corresponded to the five branches deriving from the oldest cell (Figure 5D). We found that PEG10 and SLC3A1 are the marker genes of cancer cells in the earliest stage or cancer stem-like cells in ovarian cancer; C0, one of the two malignant tumor clusters, includes these cancer stem-like cells.

### 3.4. C4 Cluster Is Associated with Decreasing Tumor-Infiltrating CTL Population

**Figure 6:**
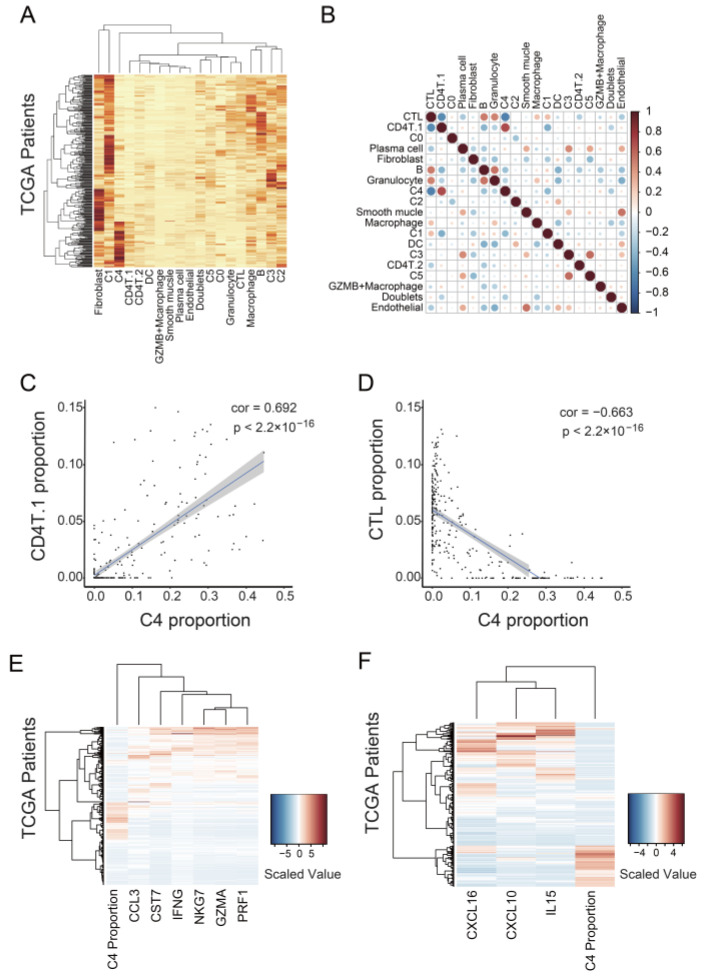
Association of the C4 cluster with CTL suppression in tumors. (**A**) Heatmap of Seurat cluster proportions in 307 samples from TCGA. The proportions of cells were calculated using CIBERSORTx. (**B**) Correlation plot of the proportions of the Seurat clusters. A strong negative correlation existed between the proportions of the C4 cluster and CTLs. (**C**) Scatter plot showing the proportion of the C4 cluster versus that of CD4T.1 for 307 TCGA samples. (**D**) Scatter plot showing the proportion of the C4 cluster versus that of CTL for 307 TCGA samples. (**E**) Heatmap showing the expression level of T-cell cytotoxicity-representing genes for 307 TCGA samples. (**F**) Heatmap showing gene expression level of chemokines and a cytokine associated with CTL migration and survival for 307 TCGA samples.

Since the C4 cluster did not have malignant properties itself in GSEA, CNV analysis, or trajectory analysis (Figure 2B, Figure 4D and Figure 5D), we examined whether the C4 cluster promoted tumor progression by affecting other cells in the tumor. We examined the relationship between C4 and other cells by classifying 307 TCGA samples into the 19 Seurat clusters, except for the Unknown cluster, with CIBERSORTx (Figure 6A). A high proportion of the C0 cluster was significantly associated with short disease-free survival (Supplementary Figure S9), consistent with the result indicating that high expression of the marker genes for C0 or C4 was significantly associated with poor prognosis (Figure 2A). However, we did not detect a significant difference between a high and low proportion of C4 in survival period (Supplementary Figure S9). The difference may be due to the difference in sample size (307 samples for Supplementary Figure S9 vs. 536 samples for Figure 2A). Of all the correlations between the clusters, we detected the strongest positive correlation between C4 and CD4T.1 (cor. = 0.692, *p* < 2.2 × 10^−16^; Figure 6B,C). CD4T.1 included Tregs expressing CD4 and FOXP3 (Supplementary Figure S1E). In addition, we detected the strongest negative correlation between C4 and CTL (cor. = −0.663, *p* < 2.2 × 10^−16^; Figure 6B,D). Tregs suppress anti-tumor immunity [48], and tumor-infiltrating CTLs are important for a good prognosis in ovarian cancer [49]. Therefore, these data suggest that the C4 cluster is involved in the suppression of tumor-infiltrating CTLs.

We further examined whether the C4 cluster is related to the decrease in the CTL population by analyzing the gene expression level in samples with high proportions of the C4 cluster. We used the data of 307 TCGA samples and calculated the cell proportions using CIBERSORTx. We first examined the expression level of T-cell cytotoxicity-representing genes, including CCL3, CST7, IFNG, NKG7, GZMA, and PRF1. Patients with a high proportion of C4 mostly expressed lower levels of the aforementioned six genes (Figure 6E). Furthermore, we found three cytokine-encoding genes, CXCL10, CXCL16, and IL15, among the genes negatively correlated with the proportion of the C4 cluster (Figure 6F). CXCL10 [50] and CXCL16 [51] are associated with CTL migration and trafficking, whereas IL15 promotes the survival and activation of CTLs [52,53]. These data support the finding that the C4 cluster is associated with the decrease in the tumor-infiltrating CTL count.

**Figure 7:**
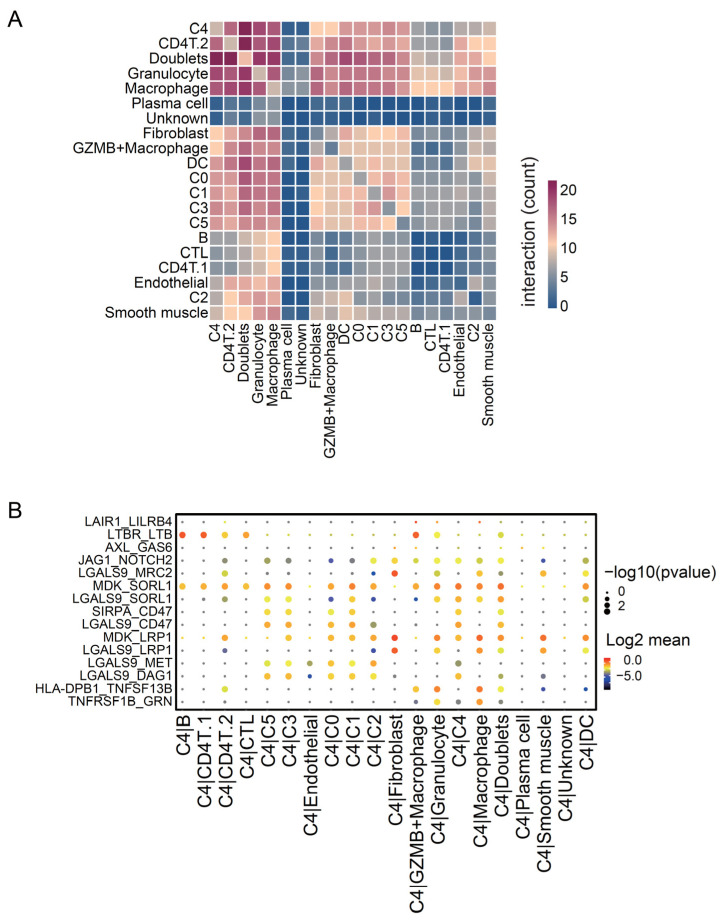
Cell–cell interaction levels between the Seurat clusters. (**A**) Heatmap of molecular interaction levels between Seurat clusters, calculated using CellPhoneDB. (**B**) Dot plot of significant ligand–receptor couples between the C4 cluster and other clusters. Dot color represents the −log10 (*p*-value), and dot size represents the log2 (mean interaction level).

To further study the relationship between C4 and other cells, we investigated which cells interacted with the C4 cluster and which molecules mediated the interaction. We quantified the interaction level between clusters using CellphoneDB. The C4 cluster interacts more strongly with immune cell clusters, including CD4T.2, macrophages, granulocytes, and macrophage-like-cancer, than other tumor clusters (Figure 7A). We also depicted each interaction level of the significant ligand–receptor couples between C4 and the other clusters (Figure 7B). Among the genes involved in the significant interactions, GAS6 and LGALS9 were expressed at higher levels in C4 than in the other tumor clusters (Supplementary Figure S10). Galectin-9, encoded by LGALS9, is known to interact with CD40 on T cells, preventing their proliferation and inducing cell death [54]. Moreover, it shifts monocytes (THP1) from the M1 to M2 polarization state [55]. Furthermore, GAS6 interacts with AXL, expressed by myeloid cells, and decreases CTL infiltration [56]. These results imply that the C4 cluster interacts with immune cells via specific molecules associated with CTL suppression and thereby decreases tumor-infiltrating CTL population and leads to poor prognosis.

## 4. Discussion

### 4.1. Identification of Marker Genes for Cancer Stem-Like Cells in Ovarian Cancer

In the present study, we identified PEG10 and SLC3A1 as the marker genes of ovarian cancer-initiating cells. PEG10 is expressed mainly in the placenta [57], and has already been identified as a gene highly associated with ovarian cancer stem cells [58]. SLC3A1 is a cysteine carrier, expressed mainly in the epithelial cells of intestinal mucosa and renal tubule [59]. SLC3A1 increases GSH expression and activates AKT, promoting breast cancer tumorigenesis. SLC3A1 may also be associated with tumorigenesis or recurrence in ovarian cancer. Regarding conventional markers of cancer stem cells, CD133, CD44, KIT, and ALDH1A1 [47] exhibited similar expression in all the tumor clusters. However, many marker genes used to identify cancer stem cells are specific to cancer types and cannot be applied to ovarian cancer [60]. In addition, these markers are often used together. Therefore, PEG10 and SLC3A1 could be used to narrow down cancer stem cells in ovarian cancer. In particular, since SLC3A1 exists in plasma membrane and exhibits low expression in normal ovarian cancer, it may be helpful as a new cell surface marker of ovarian cancer stem cells.

C0 included cancer-initiating cells expressing PEG10 and SLC3A1, while C4, specifically expressing CA125, appeared later and was derived from the oldest cells. CA125 is the most common biomarker for ovarian cancer, and its level increases in most ovarian cancers [61]. However, CA125 has lower sensitivity in the early stage of ovarian cancer [5]. Our findings indicate that it is difficult to detect CA125 in the earliest stage of ovarian cancer because cancer cells expressing CA125 differentiate as the tumor progresses. In order to identify ovarian cancer as early as possible, new biomarkers need to be explored, with a focus on cancer cells existing in the earliest stage.

### 4.2. C4 Cluster Is a New Subtype Associated with Poor Prognosis in Ovarian Cancer

We compared the C4 cluster with CAFs, which represent the main cell type in heterogeneous tumors and affect therapeutic resistance [62]. CAFs increase the intracellular GSH level and contribute to platinum resistance in ovarian cancer [63]. CAFs also suppress NK cells by gathering M2 macrophages in colorectal cancer [64]. The C4 cluster, associated with CTL suppression, expressed genes related to extracellular vesicles, one of the CAF biomarkers [62]. In addition, the C4 cluster interacted with immune cells via GAS6, which is expressed by CAF and is associated with CTL suppression [56]. However, the C4 cluster also expressed typical epithelial marker genes, including EPCAM, and did not express CAF marker genes, including ACTA2 and VIM. Thus, the C4 cluster is a subpopulation different from CAF, unless it differentiates into CAF through epithelial–mesenchymal transition (EMT) [62].

Next, we compared the C4 cluster with another malignant subpopulation in epithelial ovarian cancer. Zhao et al. (2021) found that cancer subtypes expressing IFI6 are associated with poor prognosis and that IFI6 plays a key role in cisplatin resistance [15]. Moreover, Quinn et al. (2021) found that IFI6 is a metastasis-associated gene in lung cancer [65]. In the present study, all the tumor clusters expressed IFI6 to a similar extent. Possibly, in this study, cancer cells were clustered differently from the previous study, and we found a new subtype associated with poor prognosis.

Although patients with high z-scores of C0 marker genes showed poorer prognoses than those with low scores, the high proportion of C4 was not significantly associated with poor prognosis. The difference may be caused by the different sample sizes between the two cohorts: microarray expression data of 536 patients for calculating z-scores and sequence data of 307 samples, which can be applied for CIBERSORTx analysis to calculate cell proportions. Since the *p*-value tends to be smaller as larger samples are collected, we may find a significant association between the proportion of C4 and the prognosis of patients if we collect as many samples as we used for calculating z-scores.

The C4 cluster was associated with the suppression of tumor-infiltrating CTLs. However, we detected a greater interaction between the C4 cluster and myeloid cells than that between the C4 cluster and CTLs. One possible reason is that the GAS6/AXL signaling pathway detected through cell interaction analysis was associated with CTL suppression. Interaction between GAS6 expressed by CAFs and AXL expressed by myeloid cells changes the interaction levels of PD-L1 and MHC-I, increases the secretion of cytokines involved in immune suppression, and decreases the tumor-infiltrating CTL population [56]. GAS6 expressed by the C4 cluster as well as CAFs may activate the GAS6/AXL pathway, suppressing CTLs. Samples with more cells belonging to the C4 cluster tended to have more Tregs, and they may also suppress CTLs. Another possibility is that the C4 cluster interacts with CTL and leads to its apoptosis. Both LGALS9 and LCN2, the marker genes of the C4 cluster, induce CTL apoptosis [54,66]. Thus, CTLs interacting with such molecules would die out. As a result, only the CTL subpopulation not interacting with the C4 cluster remain, making it difficult to detect the interaction between the C4 cluster and CTLs. In summary, the C4 cluster may be associated with CTL suppression by activating the GAS6/AXL pathway and promoting CTL apoptosis.

### 4.3. Limitations

Similarly to other markers of cancer stem cells, the mechanism by which cancer stem-like cells express PEG10 and SLC3A1 remains unknown. This mechanism needs to be confirmed to better understand cancer stem-like cells in ovarian cancer.

Although we found that the C4 cluster is associated with poor prognosis, it was impossible to isolate or culture only this cluster, keeping its properties intact. Therefore, we could not observe ovarian cancer progression or recurrence with or without the C4 cluster alone. Moreover, it is impossible to determine whether the C4 cluster specifically affects immune cells and causes CTL suppression without isolating the C4 cluster. New techniques are needed to confirm that the C4 cluster is the deciding factor for CTL suppression in ovarian cancer.

## 5. Conclusions

We classified epithelial cancer cells in ovarian cancer and identified malignant subtypes associated with poor prognosis. We found that one malignant population included cancer-initiating cells. SLC3A1 can be considered as a cell surface marker of cancer-initiating cells. The other malignant population was associated with the suppression of tumor-infiltrating CTLs. Blocking the interaction between this malignant population and immune cells could be a new target of treatment for patients with ovarian cancer. In the future, new techniques developed to classify cancer cells in vivo can be used in combination with scRNA-seq to examine both the gene expression levels and distribution of each subtype, leading to a better understanding of tumor heterogeneity.

## Data Availability

Sequencing data were deposited to DRA012826 and DRA012827 in the DNA Data Bank of Japan (DDBJ). The processed data in the Seurat object were deposited to 10.25739/xdex-q387 in CyVerse. The analyzed data in the study are available from the corresponding author upon reasonable request.

## Supplementary Materials

The following supporting information can be downloaded at: https://www.mdpi.com/article/10.3390/cancers14153580/s1, Figure S1: Identification of cell types for non-tumor clusters; Figure S2: Gene Set Enrichment Analysis (GSEA) of tumor clusters about Gene Ontology (GO) related to chemosensitivity; Figure S3: Kaplan–Meier plots for the clusters not associated with poor prognosis; Figure S4: All tumor clusters express the genes related to known malignant cell populations to a similar extent; Figure S5: Cell populations like C0 and C4 are identified in another single cell RNAseq dataset of ovarian cancer; Figure S6: Gene Set Variation Analysis (GSVA) for bulk samples of tumor or ascites; Figure S7: Copy number variation (CNV) analysis by patients; Figure S8: All tumor clusters express the common marker genes of cancer stem cells to a similar extent; Figure S9: Prognosis of patients with a high proportion of C0 or C4; Figure S10: C4 cluster expresses the genes associated with CTL suppression; Table S1: Clinical information of patients used for scRNAseq; Table S2: Clinical information of patients used for bulk RNAseq.
