# Supplementary material for: Identification of Malignant Cell Populations Associated with Poor Prognosis in High-Grade Serous Ovarian Cancer Using Single-Cell RNA Sequencing
Source: Cancers (Basel). 2022 Jul 22;14(15):3580. doi: 10.3390/cancers14153580 (PMC9331511; doi:10.3390/cancers14153580)
Supplement: Supplementary file 1 [file cancers-14-03580-s001.zip › cancers-1773044-supplementary.pdf]

SUPPLEMENTARY DATA

Identification of malignant cell populations associated with poor prognosis in high-grade serous ovarian cancer using single-cell RNA sequencing

Naoki Sumitani, Kyoso Ishida, Kenjiro Sawada, Tadashi Kimura, Yasufumi Kaneda, and Keisuke Nimura

Supplementary Figure Legends

Supplementary Figure S1. Identification of cell types for non-tumor clusters

(**A**) UMAP of 33,591 ovarian cancer cells, colored by patients. (**B**) Violin plot showing the proportion of mitochondrial genes in Seurat clusters. (**C**) Violin plots showing the marker gene expression level in Seurat clusters. *CD3E*, *CD79A*, *S100A8*, *ITGAM*, *ITGAX*, *FBLN2*, *SYNPO2*, and *VWF* were used as the marker genes of T cells, B cells, granulocytes, macrophages, dendritic cells (DCs), fibroblasts, smooth muscle cells (SMCs), and endothelial cells, respectively. (**D**) Violin plots illustrating the marker gene expression level for the macrophage-like cancer cluster and *GZMB*+ macrophage cluster. (**E**) Violin plots showing the expression level of the representative genes for T- and B-cell subsets.

Supplementary Figure S2. Gene Set Enrichment Analysis (GSEA) of tumor clusters about Gene Ontology (GO) related to chemosensitivity.

(**A**) GSEA enrichment plots for chemo-sensitive tumor clusters (C3 and C5), compared with the other tumor clusters (C0, C1, C2, and C4). (**B**) GSEA enrichment plot for C0, compared with the other tumor clusters (C1, C2, C3, C4, and C5). *P* value was calculated using Benjamini & Hochberg mothods.

Supplementary Figure S3. Kaplan–Meier plots for the clusters not associated with poor prognosis. Kaplan–Meier plots for all the clusters except C0, C4, Unknown, doublets, and GZMB+Macrophage, using TCGA survival data.

Supplementary Figure S4. All tumor clusters express the genes related to known malignant cell populations to a similar extent.

Violin plots showing the expression level of the representative genes for cancer-associated fibroblasts (CAFs) and malignant cell subpopulations in ovarian cancer.

Supplementary Figure S5. Cell populations like C0 and C4 are identified in another single cell RNAseq dataset of ovarian cancer.

(**A**) Violin plots showing the average expression level of the marker genes for C0 and C4 in our dataset. (**B**) Violin plots showing the average expression level of the marker genes for C0 and C4 in another single cell dataset [43].

Supplementary Figure S6. Gene Set Variation Analysis (GSVA) for bulk samples of tumor or ascites

Box plots showing the GSVA enrichment scores in 25 samples of tumor or ascites. The marker genes for C0 or C4 were used as gene sets.

Supplementary Figure S7. Copy number variation (CNV) analysis by patients
Heatmap of each patient showing CNVs on chromosomes (cols) for individual cells (rows), estimated using the infercnv R package.

Supplementary Figure S8. All tumor clusters express the common marker genes of cancer stem cells to a similar extent
Violin plots showing the expression level of the representative marker genes of cancer stem-like cells.

Supplementary Figure S9. Prognosis of patients with a high proportion of C0 or C4
Kaplan–Meier plot for the poor prognosis-associated C0 and C4 clusters, using TCGA overall survival data and disease-free survival data.

Supplementary Figure S10. C4 cluster expresses the genes associated with CTL suppression Violin plots showing the expression levels of genes that were detected in the cell-cell interaction analysis.

Supplementary Figure S1

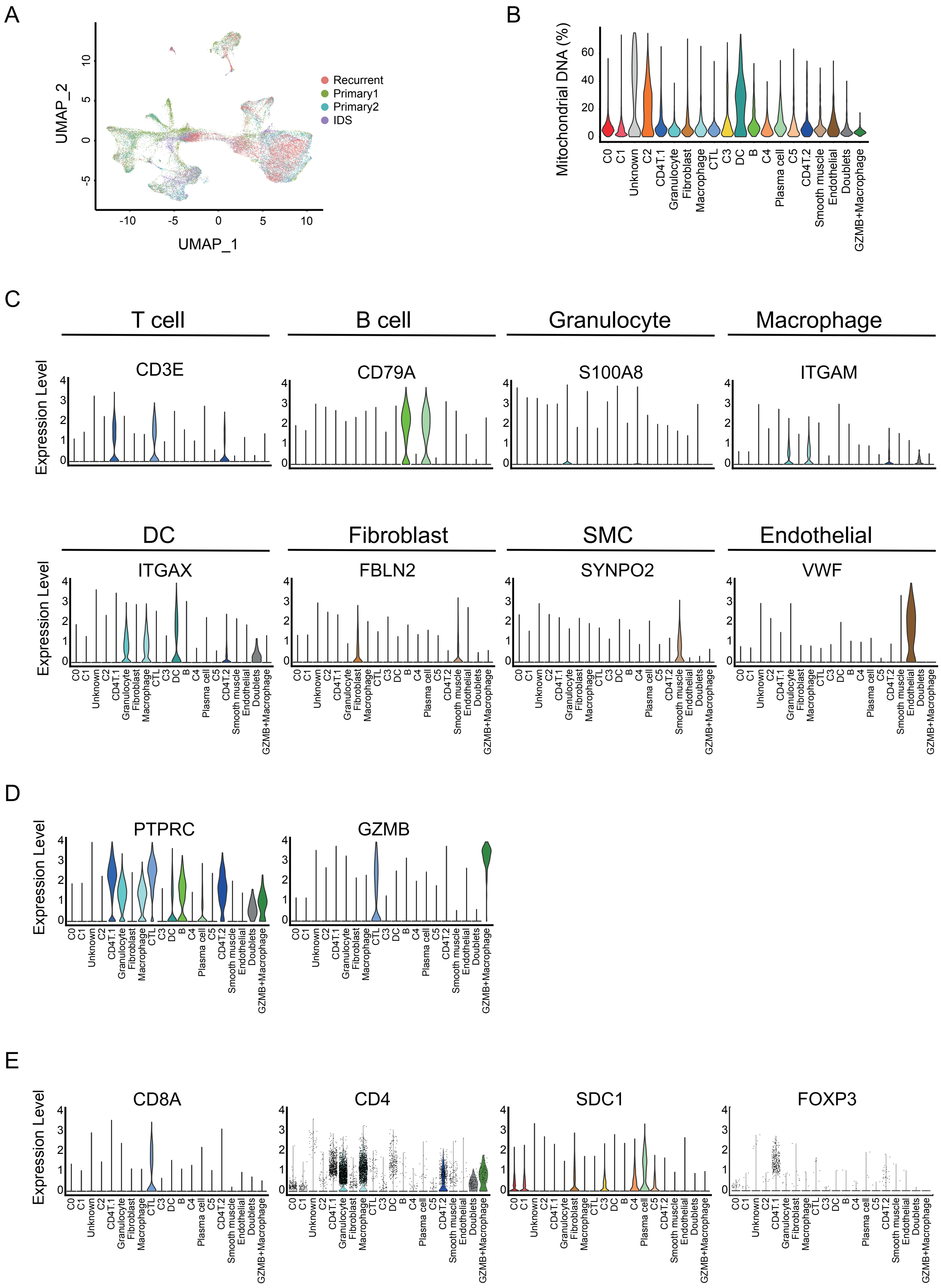

Supplementary Figure S2

A

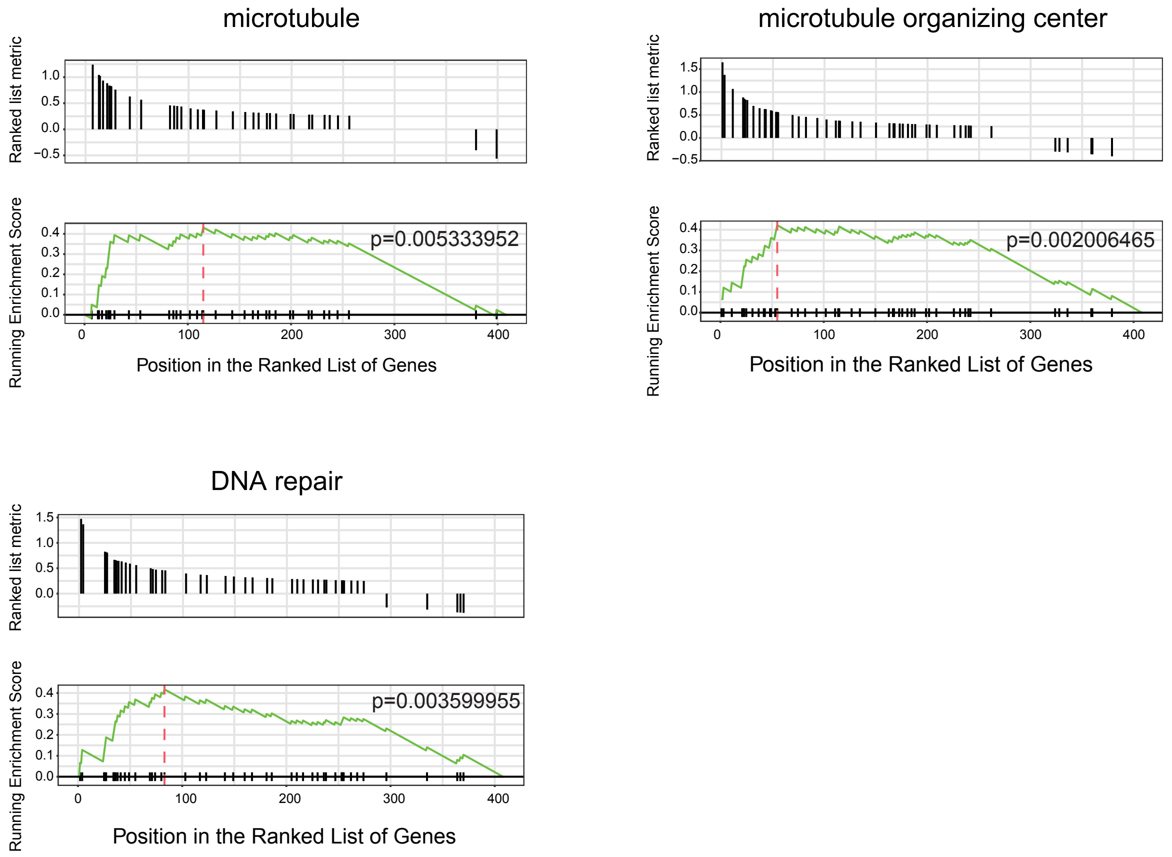

B

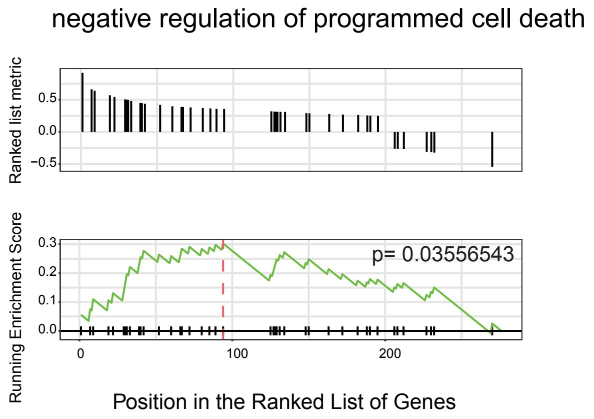

Supplementary Figure S3

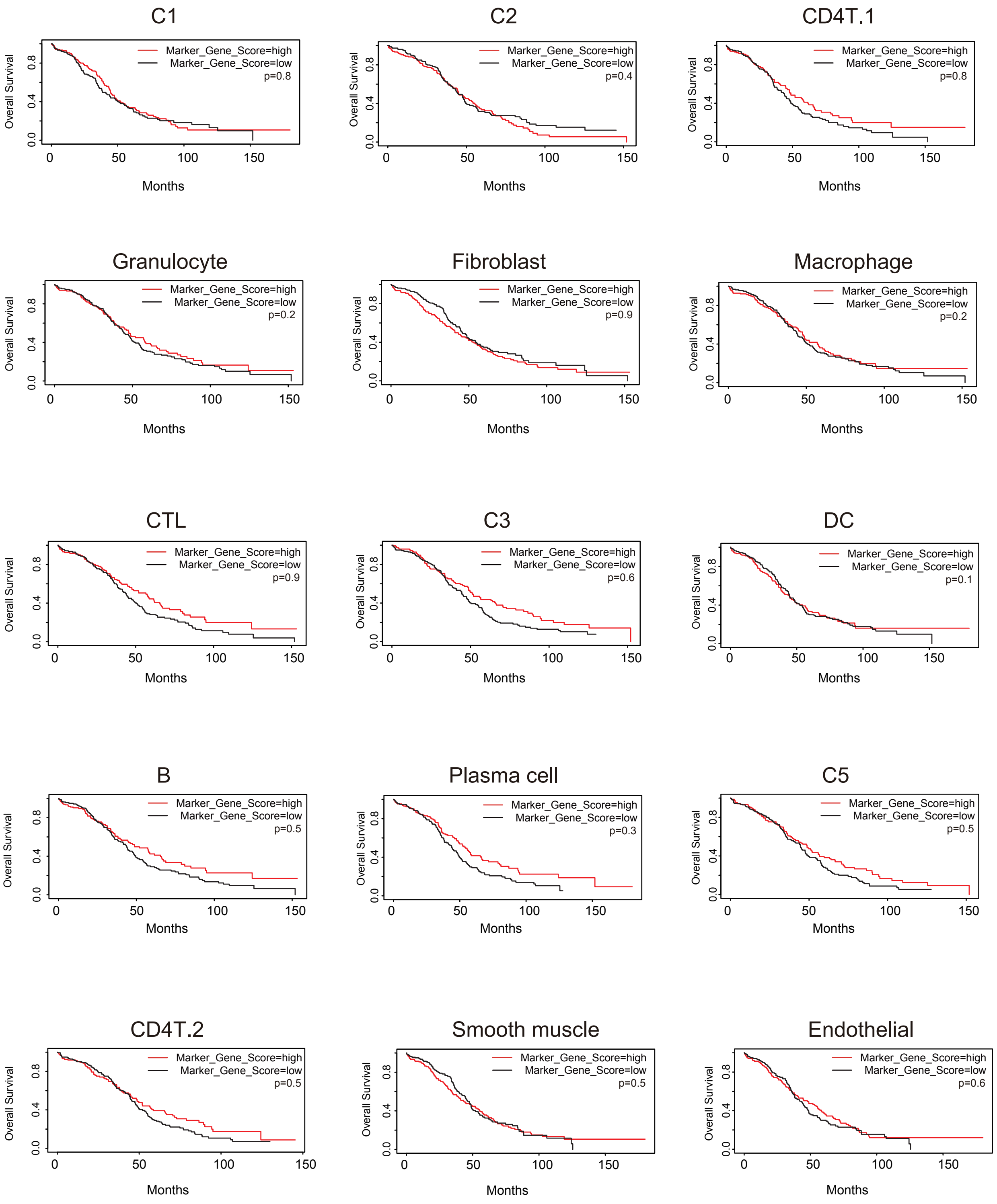

Supplementary Figure S4

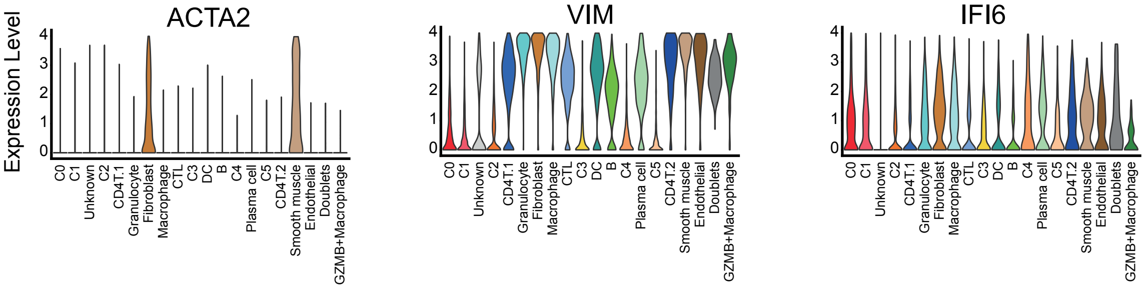

Supplementary Figure S5

A

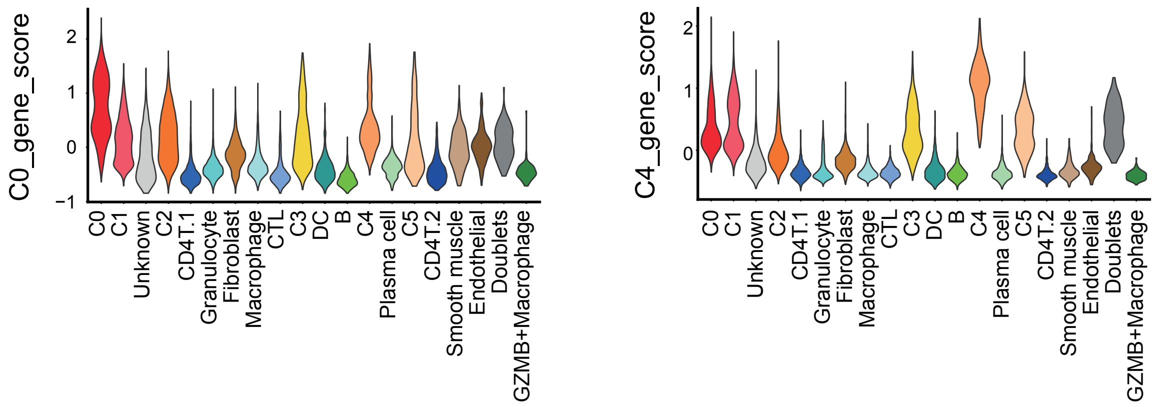

B

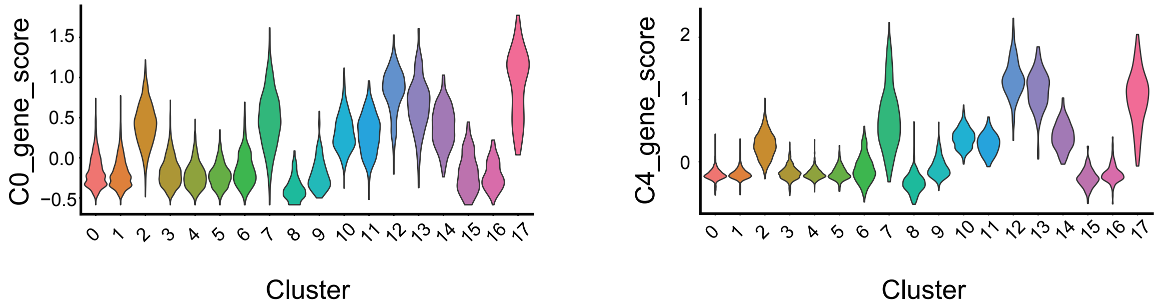

Supplementary Figure S6

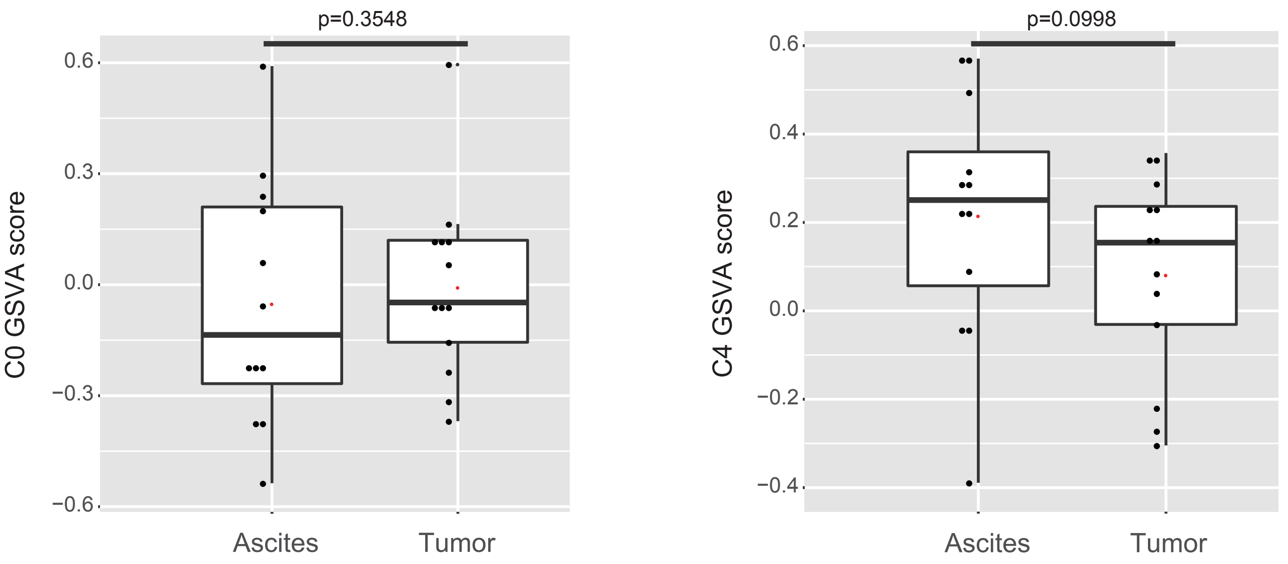

Supplementary Figure S7

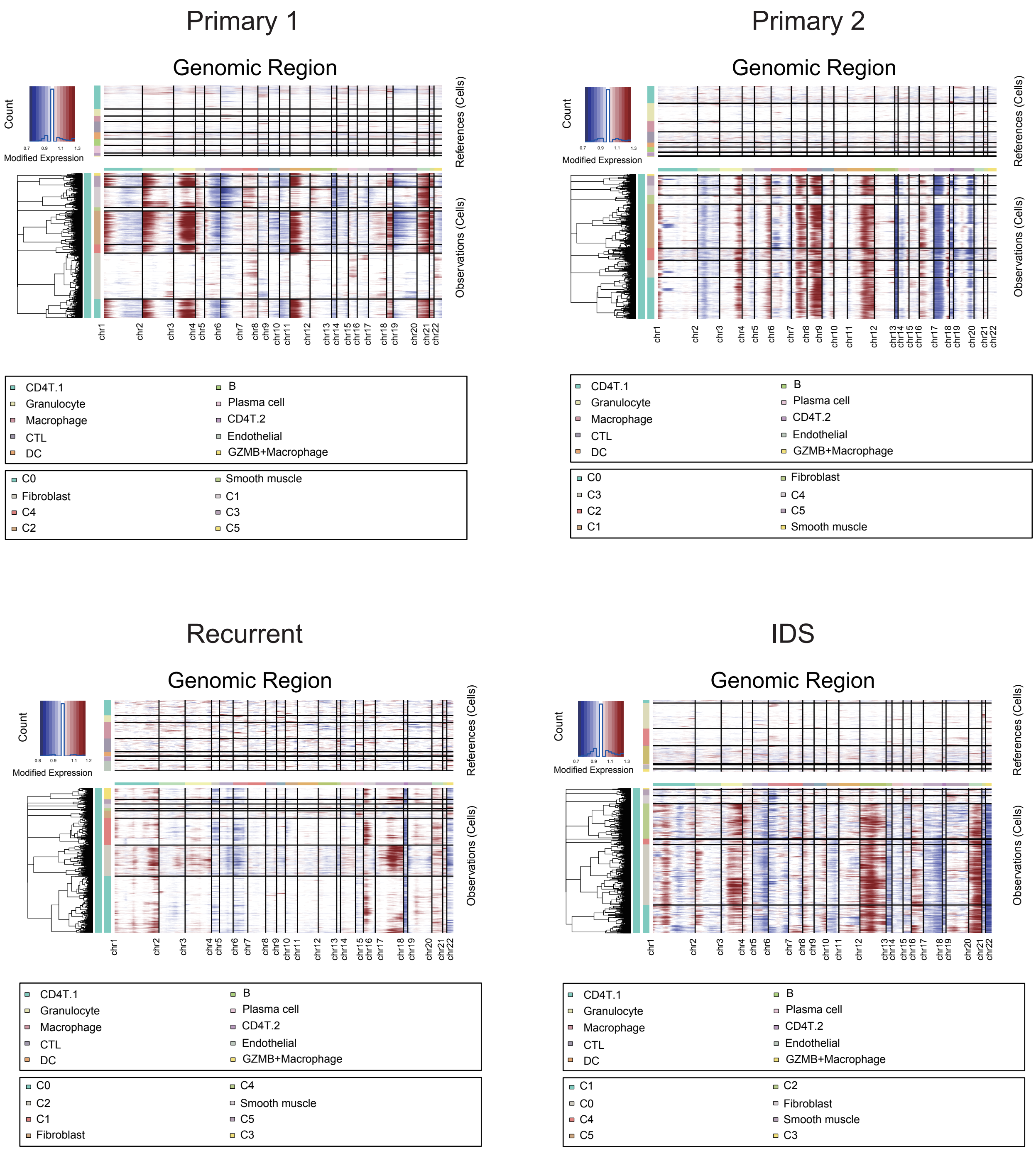

Supplementary Figure S8

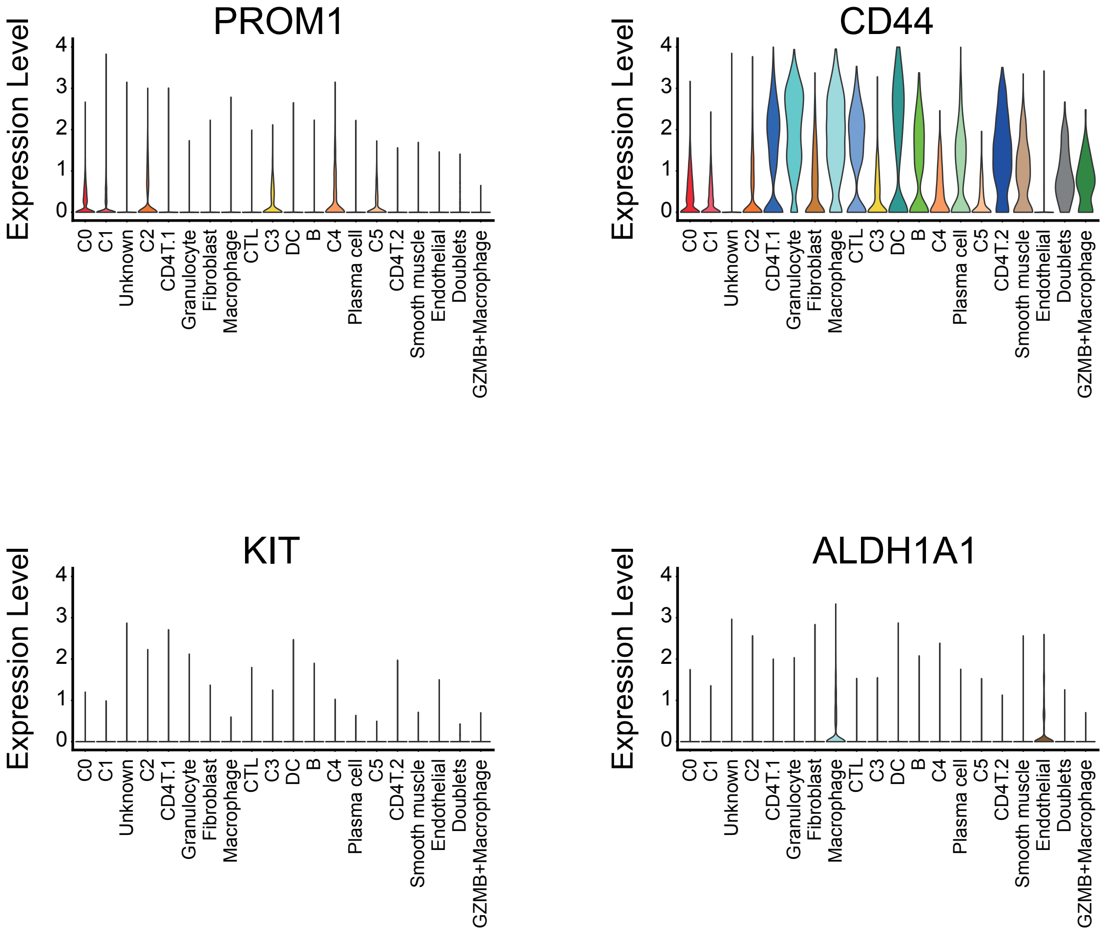

Supplementary Figure S9

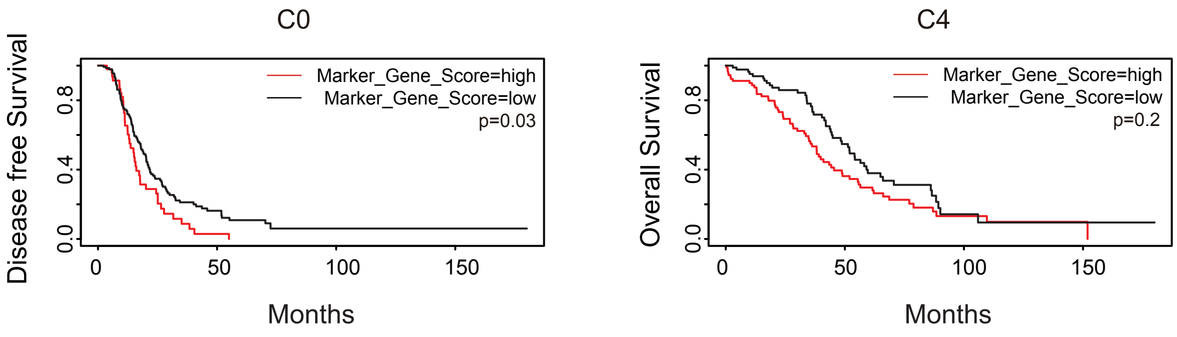

Supplementary Figure S10

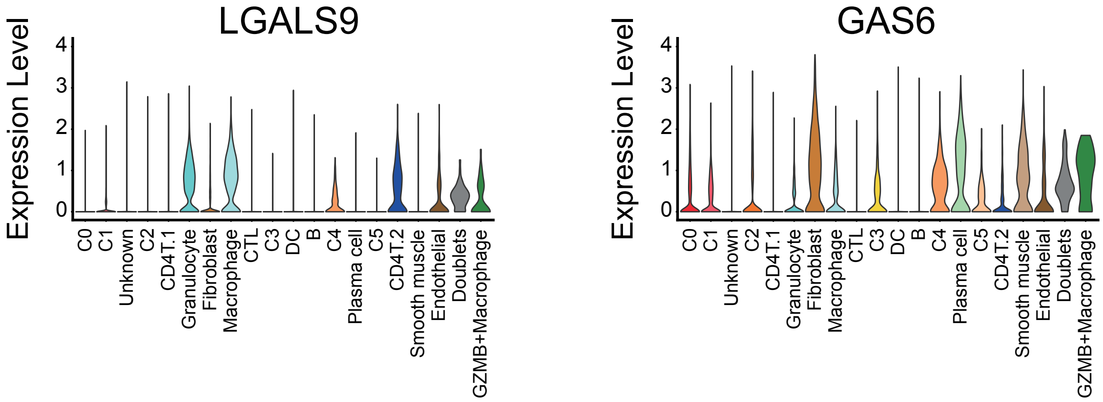

Supplementary Table S1. Clinical information of patients used for scRNAseq

Sample	Histology	BRCA status	specimen status	Chemotherapy regimen used before specimen collection
Primary 1	High-grade serous	not tested	Primary surgery (biopsy)	none
Primary 2	High-grade serous	BRCA2 mutation	Primary surgery (biopsy)	none
Recurrent	Clear cell	not tested	Recurrence	TC * 6 cycles (as adjuvant chemotherapy for primary cancer, treatment free interval > 4 years)
IDS	High-grade serous	not tested	IDS	TC * 4 cycles (as neoadjuvant chemotherapy)

TC: Paclitaxel + Carboplatin
TCB: Paclitaxel + Carboplatin + Bevacizumab

Supplementary Table S2. Clinical information of patients used for bulk RNAseq

sample	Histology	Remarks	Stage	Primary lesion
OvCa_2017_14_T00_999	HGSOC			
OvCa_2017_21_A00_999	Clear cell carcinoma		T3bN0, IIIB	Left ovary
OvCa_2017_15_T00_999	HGSOC		T3cN1bM1b, IVB	
OvCa_2017_10_A00_999	HGSOC		T1cN0M0, Ic1	
OvCa_2017_10_T00i_999	HGSOC		T1cN0M0, Ic1	Left ovary
OvCa_2017_10_T00ii_999	HGSOC		T1cN0M0, Ic1	Left ovary
OvCa_2017_04_A00_999	Clear cell carcinoma		IIIc	Right ovary
OvCa_2017_08i_A00_999	HGSOC		T3c, IIIC	Left fallopian tube
OvCa_2017_24_A00_999	HGSOC			
OvCa_2017_12_T00_999	HGSOC		T1aN0M0 IA	Left ovary
OvCa_2017_13_A00_999	HGSOC			
OvCa_2017_09_A00_999	HGSOC		T3b IIIb	
OvCa_2017_11_T00_999	Clear cell carcinoma		T1cN1M0, IIIA1(i)	Right ovary
OvCa_2017_05_A00_999	HGSOC		T3c, ypT3bN0M0	Left fallopian tube
OvCa_2017_06_A00_999	HGSOC	Reccurence		
RNA_OvK_S001_Primary_0	Endometrioid		T2cN0M0(ov; CpK=T1cN0M0)	Right ovary（＋Uterine cancer）
RNA_OvK_S010_Primary_0	Serous		T3cN0M01, IIIC	Left ovary
RNA_OvK_S008_Primary_0	Mucinous		T1a, IA	Right ovary
RNA_OvK_S011_Primary_0	HGSOC		T2bN0Mx IIB	Right ovary
RNA_OvK_S002_Primary_0	HGSOC		T3cN1aM0	Peritoneum
RNA_OvK_S006_Primary_0	Mucinous	Borderline (Low potential) malignancy	Ia1N0M0	Right ovary
RNA_OvK_S007_Primary_0	HGSOC		T1aN1bMx, IIIA1(ii)	Right fallopian tube
RNA_OvK_S005_Primary_A0	Endometrioid, Unsorted -- ascites			
RNA_OvK_S005_Primary_0	Endometrioid, Unsorted -- cancer			
RNA_OvK_S009_Primary_0	Endometrioid, Unsorted		T1cN0M0, C1	Left ovary
